# Thyroid Cancer Detection by Ultrasound Molecular Imaging with SHP2-Targeted Perfluorocarbon Nanoparticles

**DOI:** 10.1155/2018/8710862

**Published:** 2018-03-08

**Authors:** ZhongQian Hu, Bin Yang, Tiankuan Li, Jia Li

**Affiliations:** ^1^Department of Ultrasound, Zhongda Hospital, Southeast University, Nanjing, China; ^2^Department of Ultrasound, Jinling Clinical Medical College, Nanjing Medical University, Nanjing, China

## Abstract

**Background:**

Contrast-enhanced ultrasound imaging has been widely used in the ultrasound diagnosis of a variety of tumours with high diagnostic accuracy, especially in patients with hepatic carcinoma, while its application is rarely reported in thyroid cancer. The currently used ultrasound contrast agents, microbubbles, cannot be targeted to molecular markers expressed in tumour cells due to their big size, leading to a big challenge for ultrasound molecular imaging. Phase-changeable perfluorocarbon nanoparticles may resolve the penetrability limitation of microbubbles and serve as a promising probe for ultrasound molecular imaging.

**Methods:**

65 thyroid tumour samples and 40 normal samples adjacent to thyroid cancers were determined for SHP2 expression by IHC. SHP2-targeted PLGA nanoparticles (NPs-SHP2) encapsulating perfluoropentane (PFP) were prepared with PLGA-PEG as a shell material, and their specific target-binding ability was assessed in vitro and in vivo, and the effect on the enhancement of ultrasonic imaging induced by LIFU was studied in vivo.

**Results:**

In the present study, we verified that tumour overexpression of SHP2 and other protein tyrosine phosphatases regulated several cellular processes and contributed to tumorigenesis, which could be introduced to ultrasound molecular imaging for differentiating normal from malignant thyroid diagnostic nodes. The IHC test showed remarkably high expression of SHP2 in human thyroid carcinoma specimens. In thyroid tumour xenografts in mice, the imaging signal was significantly enhanced by SHP2-targeted nanoparticles after LIFU induction.

**Conclusion:**

This study provides a basis for preclinical exploration of ultrasound molecular imaging with NPs-SHP2 for clinical thyroid nodule detection to enhance diagnostic accuracy.

## 1. Introduction

In recent years, the incidence of thyroid cancer increased significantly [1]. Conventional ultrasonography (US) imaging to distinguish normal from malignant thyroid tissues has better sensitivity, but the specificity is weak (58.5%) [2]. Contrast-enhanced US (CEUS) represents an important advance in US imaging and has been used as a tool in the clinic for many years to locate tumours, such as liver tumours, with good accuracy [3–6]. Nevertheless, the diagnostic validity of current CEUS methods in thyroid tumour detection is unsatisfactory, leading to many needless surgeries and biopsies. In addition, the sensitivity of CEUS, when used alone for detection of early thyroid cancer, is lower in some reports [7, 8]. Therefore, further improvement of ultrasonic diagnosis ability is very necessary for use in thyroid carcinoma scanning.

Targeted CEUS imaging using phase-shift nanoparticles as contrast agents is considered to be with a great promising tool for molecular imaging [9–11]. These nanoparticles can be labeled with specific molecular markers as ultrasound contrast agents to target tissue sites expressing these markers, resulting in a distinct signal enhancement under ultrasound imaging after LIFU exposure. By virtue of their small size (nanometres grade), the contrast nanoparticles can pass through the vessel wall and remain predominantly within the tissue. This feature makes targeted CEUS uniquely attractive as a novel molecular imaging method to detect and monitor the tumour [12–17].

Previous studies have reported that the abnormal expression of Src homology 2 domain-containing phosphotyrosine phosphatase 2 (SHP2) plays important roles in tumour occurrence and metastasis [18–20]. SHP2 is a proven oncogene; SHP2 and other PTPs regulate many diseases' progress and contribute to tumorigenesis. Mutations of PTPN11 (encoding SHP2) were found in myeloid leukaemia patients (especially childhood leukaemia patients) and some solid tumours [21, 22]. Our findings showed that SHP2 was overexpressed in thyroid tumour cell line and in tumour tissues [23].

Although monoclonal antibodies have been applied to targeted CEUS molecular imaging over ten years [24–26], it is not known whether SHP2 can serve as a new molecular marker for thyroid tumour detection using ultrasound imaging. In this study, we bound SHP2 antibody to the surface of nanoparticles to produce targeted probe for ultrasound molecular imaging on thyroid cancer. The imaging signal in tumour area was significantly enhanced by these phase-changeable nanoparticles after LIFU induction.

**Figure 1:**
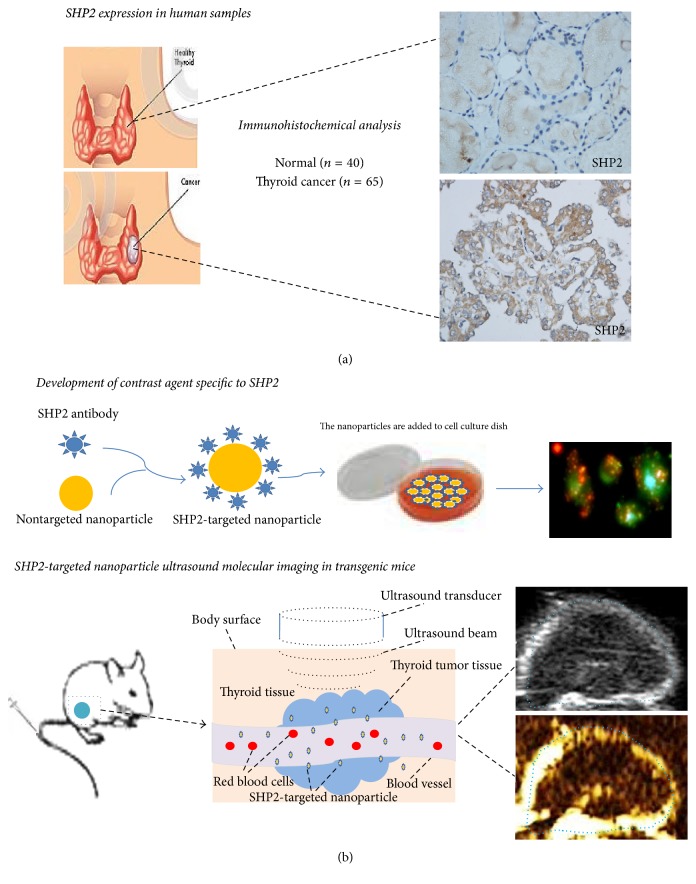
The study drawn scheme. (a) Variant expression of SHP2 in thyroid cancer was assessed on normal and malignant thyroid tissues that were collected from patients undergoing biopsy or surgical operation. (b) SHP2-targeted nanoparticles were produced and tested in vitro and vivo.

The aim of our study includes two aspects (Figure 1): (1) to compare the SHP2 expression in thyroid tumour tissue and that in normal tissue by IHC analysis and (2) to develop SHP2-targeted phase-changeable PLGA nanoparticles as a novel molecular probe for ultrasound imaging, providing a practicable method for thyroid cancer detection.

## 2. Materials and Methods

See (Figure 1) the experiment design scheme.

### 2.1. Selection of Human Thyroid Tissue Samples

In the present study, 65 human thyroid cancer samples and 40 normal thyroid samples were collected at the Department of Pathology for retrospective comparison (Table 1). The thyroid samples were processed into thyroid tissue microarray using standard protocols and the experiment methods of IHC analysis of SHP2 expression in thyroid tissue and cell culture experiments were the same as those in our previous reports [23, 27].

### 2.2. Preparation of SHP2-Targeted PLGA Nanoparticles

PFP/PLGA-PEG nanoparticles were prepared by double emulsion solvent evaporation method. In brief, 100 mg of PLGA was dissolved in 2 mL of trichloromethane and added with 200 *μ*l of PFP (Sigma-Aldrich Chemical Co., USA); the primary emulsion was obtained by an ultrasonic probe (VCX-130, Sonics & Materials Inc., USA). The precipitate was collected by centrifugation, washed, and resuspended with PBS (pH = 6.0) to disperse nanoparticles concentration of 10 mg/mL. The coupling activator EDC (0.1 mL, 50 mg/mL) and NHS (0.1 mL, 50 mg/mL) were dissolved in 1 mL of double distilled water and then were mixed with the PLGA nanoparticle solution. After shocking the reaction at room temperature for 2 h, the sample was dispersed again in deionized water after multiple centrifugal separations and purifications in the appropriate amount of PBS (pH = 8.0); 200 *µ*l of SHP2 antibody solution was added, followed by the reaction at room temperature for 2 h. After washing, the sample was redispersed in a suitable amount of PBS. Then, 500 *μ*l of polyethylenimine dissolved in 2 ml of deionized water was added to this solution, and dilute hydrochloric acid was added as necessary to adjust the pH to 8.0. After shocking the homogeneous reaction at room temperature for 2 h, the solution was purified by centrifugation and redispersed in PBS (pH = 8.0). Then, 30 nmol DOTA-NHS was added to the solution and reacted for 2 h; after washing, the product was dispersed in PBS solution (pH = 8.0).

### 2.3. In Vitro Binding Specificity of SHP2-Targeted Nanoparticles

The cells were seeded in six-well plates for 24 h until 50% confluence was reached. The cell membrane was then stained with DiO (Keygen, China). The cells were treated with either NPs-SHP2 or control nontargeted nanoparticles (NPs-Control) at 37°C for 30 minutes; both groups of nanoparticles were stained with Dil (Keygen, China) in advance. The cells were washed and fixed and then imaged.

### 2.4. Mouse Model

Animal protocols were approved by the Animal Studies Core Facility at the Chongqing Medical University. Subcutaneous human thyroid cancer xenograft tumours were established in the right flank region of 10 female 4-week-old nude mice (*n* = 10) by subcutaneous injection of 2 × 10^6^ SW579 cells in 100 *μ*L of PBS. Tumours were allowed to grow to a mean maximum diameter of 10 mm (range: 8–10 mm). The mice were examined 3 weeks after tumour inoculation.

### 2.5. SHP2-Targeted Contrast-Enhanced Ultrasound Imaging of Mice

The mice bearing thyroid tumours were imaged. Each mouse was injected with 0.1 ml of NPs-SHP2 into the caudal vein, and the same amount of NPs-Control was used in the control groups. Images of the signal from adherent nanoparticles appeared as green maps on contrast-mode images, which were automatically calculated using Vevo CQ software (VisualSonics). The colour map scale used was the same for all images.

### 2.6. Analysis of Mouse Tumour Imaging Data

Imaging data of all mice were analysed offline using software. All data were analysed in a blinded manner. Regions of representing signal were drawn over as colour maps in thyroid tumour contrast images, and quantification of the imaging signal from attached nanoparticles was assessed by calculating imaging signals [19, 27, 28].

### 2.7. Statistical Analysis

All data are expressed as the means ± SD. Means were compared using one-way analysis of variance (ANOVA) and Student's *t*-test, and *P* values < 0.05 were considered statistically significant.

## 3. Results

### 3.1. SHP2 Expression in Human Thyroid Cancer Tissues

**Figure 2:**
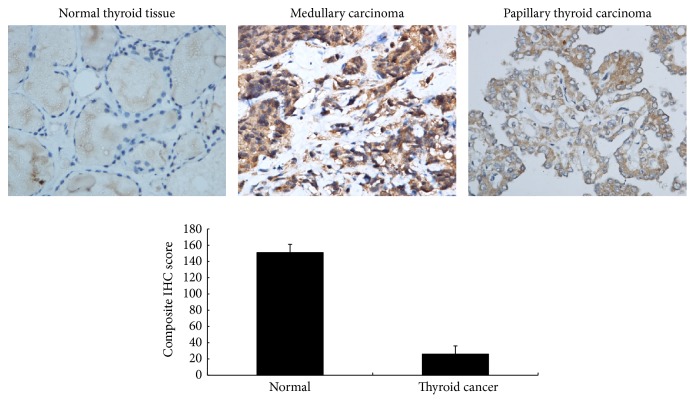
SHP2 expression in human thyroid tissues. It shows emblematic dyeing results from normal thyroid and from thyroid cancer tissues of various types. The graph displays composite IHC scores for SHP2-dyed normal and thyroid cancer tissues. *P* < 0.001.

To investigate the roles of SHP2 in thyroid cancer, we compared the expression of SHP2 in thyroid cancers and normal thyroid tissues through standard immunohistochemistry (IHC); it was performed on thyroid tissues representing 65 thyroid tumours (Table 1). In the 65 samples that were processed into a thyroid cancer TMA, the positive signal of SHP2 in the cytoplasm and nuclei of the thyroid tumour cells was markedly stronger (*P* < 0.001) compared to that in the surrounding normal tissue (Figure 2). The mean composite IHC score of tumours was also increased.

### 3.2. In Vitro Binding Specificity of SHP2-Targeted Nanoparticles

**Figure 3:**
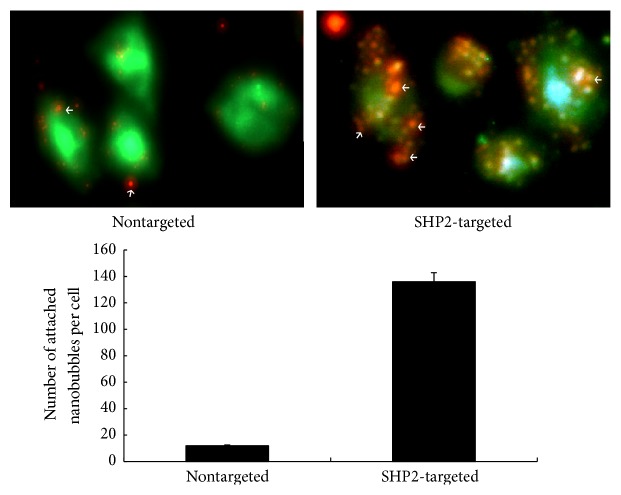
The SHP2-targeted nanoparticles binding specificity test. Representative results from in vitro experiments after exposure to SHP2-targeted and nontargeted nanoparticles. Note the specific attachment of SHP2-targeted nanoparticles and the substantial binding inhibition following the administration of nontargeted nanoparticles. Nanoparticles are shown as red dots. *P* < 0.01.

NPs-SHP2 and NPs-Control were prepared, the particle diameter of the nanoparticles is centralized and distributed as a single peak with a mean diameter of 531.2 ± 13.5 nm, an electric potential of −14.0 mV (NPs-Control), with a mean diameter of 535.7 ± 14.7 nm, and an electric potential of −13.7 mV (NPs-SHP2), and the targeting ability to SHP2 was checked by in vitro experiments.  Figure 3 shows the NPs-SHP2 and NPs-Control targeting to SW579 cells in six-well plates. The number of NPs-SHP2 attached per cell was notably higher (*P* < 0.001) than that of NPs-Control.

### 3.3. NPs-SHP2 Ultrasound Imaging In Vivo Experiment

**Figure 4:**
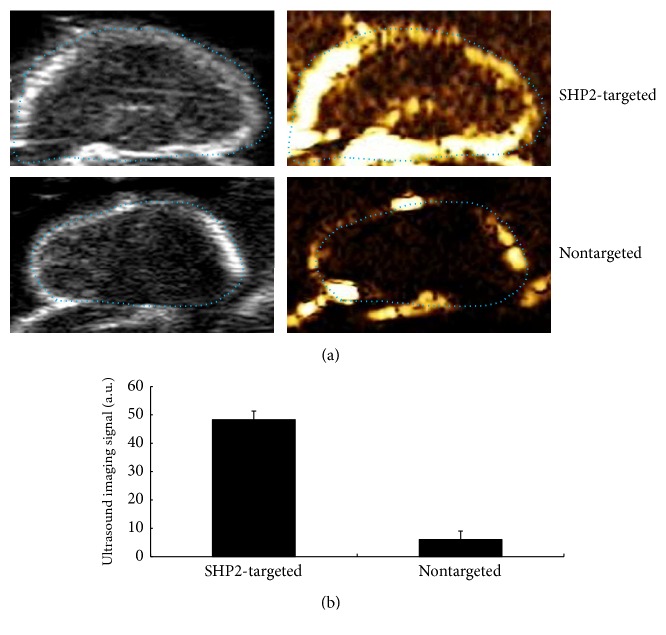
Target molecular imaging in vivo experiments after LIFU irradiation 1.40 w/cm^2^ for 20 min. (a) Different ultrasound images mode following the injection of SHP2-signed contrast nanoparticles showing a high signal in thyroid tumour and showing only background signal when using nontargeted contrast nanoparticles. (b) A bar graph summarizing the quantitative signal obtained using ultrasound imaging with SHP2-signed and nontargeted nanoparticles in a thyroid cancer mouse model; a significantly increased imaging signal was observed in the SHP2-targeted nanoparticles compared to the nontargeted nanoparticles in the tumour tissue. *P* < 0.001.

The ultrasound molecular imaging with NPs-SHP2 was performed in thyroid tumour bearing mice. After NPs-SHP2 injection followed by LIFU irradiation (1.40 w/cm^2^ for 20 min), the ultrasound signal in tumour area was significantly increased, while no enhancement was found after NPs-Control administrated as shown in Figure 4. There was a significant difference between NPs-SHP2 (48.32 ± 2.9 a.u.) and NPs-Control (6.03 ± 1.6 a.u.) groups (*P* < 0.001).

## 4. Discussion

SHP2, which is encoded by the PTPN11 gene in humans, is essential for multiple cellular signaling pathways that modulate cell apoptosis and motility as well as embryonic and haematopoietic cell development [18–23]. Some studies have shown that SHP2 expression of the tumour may increase the risk of metastasis in various types of cancer, including liver [23], colon [27], and breast cancers [29–31]. In thyroid cancer, the tumour expression of SHP2 was positively associated with tumour differentiation and progression. In our previous study, we also found that SHP2 expression was increased markedly in thyroid carcinoma tissue and was associated with thyroid cancer metastasis. From the previous reports, targeted CEUS imaging has been used to improve the diagnostic accuracy of ultrasound for the early detection of pancreatic [10, 28], breast [11, 32], and ovarian cancers [33]. However, whether SHP2 may be used as a novel ultrasound imaging target for thyroid cancer imaging remains unclear.

In this study, SHP2 was confirmed to be discriminatively expressed in human thyroid tumour and normal tissues as shown by IHC, which allowed it to be a potential marker for the identification of thyroid tumour by molecular ultrasound imaging with higher diagnostic accuracy.

SHP2-targeted (NPs-SHP2) nanoparticles were then designed and their binding specificity was investigated by in vitro and in vivo experiments. The results of cell-based experiments showed high affinity of NPs-SHP2 targeting to thyroid cancer cells. The contrast ultrasound imaging signal in thyroid tumour tissue in nude mice was strikingly higher after caudal vein injection with SHP2-targeted nanoparticles compared to that with NPs-Control injection followed by LIFU irradiation. These results together suggest that SHP2-targeted ultrasound imaging protocol should be further explored as a real-time, noninvasive, and inexpensive method for thyroid tumour detection and characterization in clinic.

In conclusion, the results of this study indicate that SHP2 is upregulated in thyroid cancer tissues compared with normal thyroid tissue obtained from surgical operation or biopsy. NPs-SHP2 had high specificity targeting to thyroid tumour in vitro and vivo and could be activated by LIFU irradiation to enhance ultrasound molecular imaging in thyroid cancer model. Future work should aim at the development of SHP2-targeted contrast agents with clinical grade and much efforts need to be made to promote the clinical translation of ultrasound molecular imaging technique, which could improve the diagnostic accuracy of thyroid lesions by ultrasonography.

## Figures and Tables

**Table 1:** Summary table of different thyroid cancer pathologies analysed.

Histology	Subtype	Number (*n*)
Normal thyroid tissue		40
Thyroid cancer	Papillary	31
	Follicle	22
	Medullary	11
	Undifferentiated	1
